# Supplementary material for: Epidemiological Characteristics and Survival in Patients with De Novo Metastatic Prostate Cancer
Source: Cancers (Basel). 2020 Oct 3;12(10):2855. doi: 10.3390/cancers12102855 (PMC7650780; doi:10.3390/cancers12102855)
Supplement: Supplementary file 1 [file cancers-12-02855-s001.zip › cancers-958134-supplementary.docx]

Supplementary Materials: Epidemiological Characteristics and Survival in Patients with De Novo Metastatic Prostate Cancer

Carlo Cattrini Davide Soldato, Alessandra Rubagotti, Linda Zinoli, Elisa Zanardi, Paola Barboro, Carlo Messina, Elena Castro, David Olmos and Francesco Boccardo

**Table S1.** Baseline characteristics – contingency table. The contingency tables below provide the following information for each variable: the number of patients, (the expected patients) and [the chi-square statistic for each cell]. The chi-square statistic, p-value and statement of significance appear beneath the table.

| **Age** | **Cohort A** | **Cohort B** | **Cohort C** | **All patients** |
| --- | --- | --- | --- | --- |
| 15–54 | 474 (477.42) [0.02] | 970 (932.81) [1.48] | 643 (676.77) [1.69] | 2087 |
| 55–64 | 1250 (1446.44) [26.68] | 2857 (2826.14) [0.34] | 2216 (2050.42) [13.37] | 6323 |
| 65–74 | 1804 (1805.36) [0.00] | 3391 (3527.43) [5.28] | 2697 (2559.21) [7.42] | 7892 |
| 75–84 | 1862 (1623.96) [34.89] | 3268 (3172.98) [2.85] | 1969 (2302.06) [48.19] | 7099 |
| 85+ | 657 (693.82) [1.95] | 1329 (1355.64) [0.52] | 1047 (983.54) [4.09] | 3033 |
| All ages | 6047 | 11815 | 8572 | 26434 |
| The chi-square statistic is 148.7724. The *p*-value is <0.00001 | | | | |
| **Age** | **Cohort A** | **Cohort B** | **Cohort C** | **All patients** |
| <75 | 3528 (3729.22) [10.86] | 7218 (7286.38) [0.64] | 5556 (5286.40) [13.75] | 16302 |
| >=75 | 2519 (2317.78) [17.47] | 4597 (4528.62) [1.03] | 3016 (3285.60) [22.12] | 10132 |
| All ages | 6047 | 11815 | 8572 | 26434 |
| The chi-square statistic is 65.8714. The p-value is < 0.00001. | | | | |

| **Race** | **Cohort A** | **Cohort B** | **Cohort C** | **All patients** |
| --- | --- | --- | --- | --- |
| White | 3830 (3780.36) [0.65] | 7361 (7379.58) [0.05] | 5322 (5353.06) [0.18] | 16513 |
| Black | 1227 (1170.07) [2.77] | 2279 (2284.08) [0.01] | 1605 (1656.85) [1.62] | 5111 |
| Ai/An | 31 (38.92) [1.61] | 76 (75.97) [0.00] | 63 (55.11) [1.13] | 170 |
| As/Pi | 329 (339.74) [0.34] | 680 (663.19) [0.43] | 475 (481.07) [0.08] | 1484 |
| Hispanic | 614 (701.91) [11.01] | 1377 (1370.18) [0.03] | 1075 (993.91) [6.62] | 3066 |
| All races | 6031 | 11773 | 8540 | 26344 |
| The chi-square statistic is 26.5236. The p-value is .000854 | | | | |

| **Metastatic status** | **Cohort B** | **Cohort C** | **All patients** |
| --- | --- | --- | --- |
| M1a | 610 (637.79) [1.21] | 487 (459.21) [1.68] | 1097 |
| M1b | 8011 (8314.55) [11.08] | 6290 (5986.45) [15.39] | 14301 |
| M1c | 2811 (2479.66) [44.28] | 1454 (1785.34) [61.49] | 4265 |
| **All M1** | 11432 | 8231 | 19663 |
| The chi-square statistic is 135.1375. The *p*-value is < 0.00001. | | | |

**Table S2.** Multivariate model for OS in cohort B and C.

| Variables | | Number of Patients | HR | 95% CI | | *p* |
| --- | --- | --- | --- | --- | --- | --- |
|  |  |  |  | Lower | Upper |  |
| Age (years) | 15–54 | 1569 |  |  |  | <0.001 |
|  | 55–64 | 4884 | 0.98 | 0.91 | 1.05 | 0.535 |
|  | 65–74 | 5859 | 1.04 | 0.97 | 1.11 | 0.245 |
|  | 75–84 | 5018 | 1.41 | 1.32 | 1.51 | <0.001 |
|  | ≥85 | 2262 | 2.25 | 2.09 | 2.42 | <0.001 |
| Race | White | 12252 |  |  |  | <0.001 |
|  | Black | 3726 | 1.10 | 1.05 | 1.15 | <0.001 |
|  | American Indian/Alaska Native | 133 | 1.08 | 0.89 | 1.32 | 0.429 |
|  | Asian or Pacific Islander | 1113 | 0.72 | 0.67 | 0.78 | <0.001 |
|  | Hispanic | 2368 | 0.93 | 0.88 | 0.98 | 0.010 |
| Metastatic Stage | M1a  M1b  M1c | 1088  14250  4254 | 1.39  1.77 | 1.28  1.63 | 1.51  1.93 | <0.001  <0.001  <0.001 |
| Year of Diagnosis | 2004–2010  2011–2014 | 11391  8201 | 0.94 | 0.91 | 0.97 | 0.001 |

HR: Hazard ratios. 95% CI: 95% confidence intervals.

**Table S3.** Multivariate model for CSS in cohort B and C.

| **Variables** | | **Number of Patients** | **HR** | **95% CI** | | ***p*** |
| --- | --- | --- | --- | --- | --- | --- |
|  |  |  |  | Lower | Upper |  |
| Age (years) | 15-54 | 1547 |  |  |  | <0.001 |
|  | 55-64 | 4823 | 0.94 | 0.88 | 1.01 | 0.100 |
|  | 65-74 | 5760 | 0.94 | 0.88 | 1.01 | 0.104 |
|  | 75-84 | 4948 | 1.17 | 1.10 | 1.26 | <0.001 |
|  | ≥85 | 2230 | 1.74 | 1.61 | 1.89 | <0.001 |
| Race | White | 12153 |  |  |  | <0.001 |
|  | Black | 3684 | 1.06 | 1.02 | 1.11 | 0.007 |
|  | American Indian/Alaska Native | 132 | 1.07 | 0.87 | 1.32 | 0.524 |
|  | Asian or Pacific Islander | 1069 | 0.72 | 0.67 | 0.79 | <0.001 |
|  | Hispanic | 2270 | 0.95 | 0.89 | 1.00 | 0.057 |
| Metastatic Stage | M1a  M1b  M1c | 1069  14050  4189 | 1.47  1.87 | 1.35  1.71 | 1.60  2.05 | <0.001  <0.001  <0.001 |
| Year of Diagnosis | 2004-2010  2011-2014 | 11221  8087 | 0.89 | 0.85 | 0.92 | <0.001 |

HR: Hazard ratios. 95% CI: 95% confidence intervals.

| 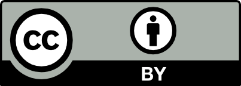 | © 2020 by the authors. Submitted for possible open access publication under the terms and conditions of the Creative Commons Attribution (CC BY) license (http://creativecommons.org/licenses/by/4.0/). |
| --- | --- |
